# Artificial intelligence and perinatology: a study on accelerated academic production- a bibliometric analysis

**DOI:** 10.3389/fmed.2025.1505450

**Published:** 2025-02-19

**Authors:** Ümran Kılınçdemir Turgut

**Affiliations:** Department of Obstetrics and Gynaecology, Perinatology, University of Health Sciences, Adana Dr. Turgut Noyan Training and Research Center, Başkent University, Adana, Türkiye

**Keywords:** deep learning, machine learning, artificial intelligence, perinatology, bibliometric analysis, fetal imaging techniques

## Abstract

**Objective:**

The main purpose of this bibliometric study is to compile the rapidly increasing articles in the field of perinatology in recent years and to shed light on the research areas where studies are concentrated.

**Materials and methods:**

This bibliometric study was conducted using the Thomson ISI Web of Science Core Collection (WOSCC) system on May 4, 2024, with specific keywords. The abstracts of 1,124 articles that met the criteria were reviewed, and 382 articles related to perinatology were evaluated. Keyword co-occurrence, co-citation of authors, and co-citation of references analyses were conducted using VOSviewer (version 1.6.19). Out of these, 121 articles with 10 or more citations were analyzed in terms of their content and categorized under the headings “Purpose of Evaluation,” “Medical Methods and Parameters Used,” “Output To Be Evaluated,” and “Fetal System or Region Being Evaluated.”

**Results:**

In this bibliometric study, it was found that the most frequently published journal among the 382 examined articles was *Medical Image Analysis*, while the journals with the most publications in the field of perinatology were *Prenatal Diagnosis* and *Ultrasound in Obstetrıcs & Gynecology.* The most commonly used keyword was “deep learning” (115/382). Among the 121 highly cited articles, the most common purpose of evaluation was “Prenatal Screening.” Artificial intelligence was most frequently used in ultrasound (59.8%) imaging, with MRI (20.5%) in second place. Among the evaluated outputs, “organ scanning” (35/121) was in first place, while “biometry” (34/121) was in second place. In terms of evaluated systems and organs, “growth screening” (35/121) was the most common, followed by the “neurological system” (33/121) and then the “cardiovascular system” (18/121).

**Conclusion:**

I has witnessed the increasing influence of artificial intelligence in the field of perinatology in recent years. This impact may mark the historic beginning of the transition to the AI era in perinatology. Milestones are being laid on the path from prenatal screening to prenatal treatment.

## Introduction

Artificial Intelligence (AI) is a broad field in computer science focused on creating systems that can perform tasks requiring human-like intelligence. Within AI, Machine Learning (ML) and Deep Learning (DL) are key areas.

Machine Learning is a branch of AI that involves developing algorithms enabling computers to learn from data and make predictions. Unlike traditional programming, where specific instructions are given, ML systems improve their performance by analyzing data. Machine learning is a field of study that gives computers the ability to learn without being explicitly programmed (1). Deep Learning is a specialized part of ML that uses complex neural networks with many layers to understand intricate patterns in data. It is explained as “Deep learning allows computational models that are composed of multiple processing layers to learn representations of data with multiple levels of abstraction” (2).

Together, AI, ML, and DL are driving significant advancements across various fields, from self-driving cars to medical diagnostics, showcasing their major impact on modern technology.

Perinatology is a branch of medicine that examines and manages complications during pregnancy and childbirth. This field deals with the assessment and treatment of high-risk pregnancies for both the mother and the fetus. Perinatologists, commonly known as maternal-fetal medicine specialists, perform various medical interventions to protect both maternal and fetal health.

In recent years, a bibliometric analysis has been prepared to highlight the increasing importance of artificial intelligence (AI) in the field of perinatology. This paper aims to explore the areas where AI has begun to be utilized within the domain of perinatology and to identify the specific objectives it focuses on.

## Materials and methods

Ethical committee approval was not sought for this study, as it was a bibliometric study and involving no human subjects.

### Study design

**FIGURE 1:**
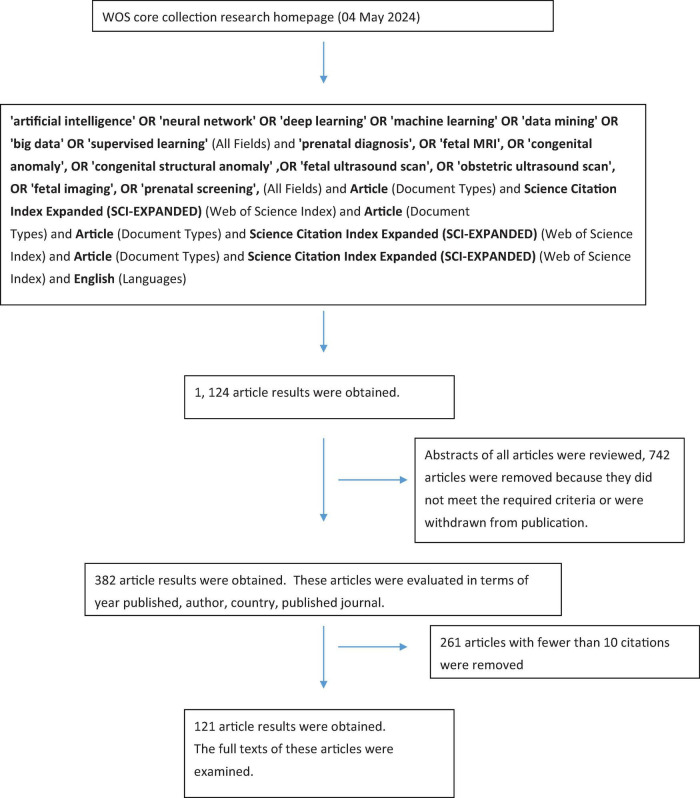
Flowchart depicting the selection and evaluation of articles.

A search was conducted on the Thomson ISI Web of Science Core Collection (WOSCC) system on May 4, 2024, using specific keywords. The keywords used were: “artificial intelligence” OR “neural network” OR “deep learning” OR “machine learning” OR “data mining” OR “big data” OR “supervised learning” (All Fields) AND “prenatal diagnosis,” OR “fetal MRI,” OR “congenital anomaly,” OR “congenital structural anomaly,” OR “fetal ultrasound scan,” OR “obstetric ultrasound scan,” OR “fetal imaging,” OR “prenatal screening” (All Fields). The search was limited to articles (Document Types) and the Science Citation Index Expanded (SCI-EXPANDED) (Web of Science Index), and only articles in English (Languages) were considered (Figure 1).

The search yielded 1,124 articles. All articles relevant to the screening results were evaluated by perinatology specialist (Ü.K.T.). The abstracts of these articles were reviewed, and those that were irrelevant, did not meet the search criteria, or had been retracted were excluded. A total of 382 articles met the criteria.

### Data analysis

VOSviewer (version 1.6.19) is a bibliometric tool that supports quantitative literature analysis. This tool allows for the creation of co-occurrence, co-citation, and co-authorship maps based on a defined dataset. Keywords were analyzed using co-occurrence analysis, which examines the frequency with which pairs of keywords appear together in the same article. For citation analysis, references, cited authors, and journals were selected. When a third article cites two other articles in its reference list, those two articles have a co-citation relationship. Authors and institutions were assessed through collaborative network analysis.

### Evaluation of articles with 10 or more citations

The full texts of 121 articles with 10 or more citations were evaluated by perinatology specialist (U.K.T). They were categorized based on their content.

#### Categorization of variables

While categorizing the articles, four key questions were addressed.

*1- Did the purpose of using artificial intelligence in the articles focus on one of the areas of screening, diagnosis, or treatment?” Purpose of Evaluation.”*

A total of 121 articles were re-evaluated and categorized based on various aspects. In terms of the *purpose of evaluation*, they were divided into three categories. If the study aimed to assess a normal condition and determine risk, it was classified as “screening.” If the purpose was to diagnose a specific disease, it was categorized as “diagnosis.” If the research involved the use of a method within the context of a treatment, it was classified as “treatment.”

*2-On which medical method or parameter was artificial intelligence integrated and applied? “Medical Methods and Parameters Used.”*

When evaluated in terms of the *medical methods and parameters used*, the articles were categorized based on the following techniques: ultrasound, magnetic resonance imaging (MRI), genetic testing, electronic fetal monitoring (NST), the combination of physical examination and patient history, laboratory blood parameters, the combination of physical examination and laboratory results, and fetoscopic surgery.

*3- What medical data/outputs were evaluated using artificial intelligence? “Output To Be Evaluated.”*

In cases where measurements such as Biparietal Diameter (BPD), Head Circumference (HC), Abdominal Circumference (AC), Femur Diaphysis Length (FDL), or Crown-Rump Length (CRL) were utilized, they were classified under “biometry.” If the focus was on assessing specific fetal anomalies, they were categorized as “congenital anomaly.” The identification of genetic disorders fell under the category of “genetic anomaly.” Evaluations aimed at recognizing placental location or pathologies were labeled as “placenta examination.” Assessments of fetal viability or wellbeing were categorized as “fetal wellbeing.” Methods involving the automatic recognition of sections taken for standard second-trimester screening, as per International Society of Ultrasound in Obstetrics and Gynecology (ISUOG) guidelines, were termed “organ screening.” Evaluations aimed at assessing pre-postnatal risks and determining prognosis for the fetus were classified as “prognosis prediction.” Finally, evaluations of fetal risks following maternal exposure to teratogenic substances were termed “teratogenicity.” Any other studies that did not fit into these categories were labeled as “other.”

*4- Which fetal systems/regions were most commonly targeted by the artificial intelligence application? “Fetal System or Region Being Evaluated.”*

The regions, systems, or diseases focused on in the study were categorized accordingly. If the study pertained to fetal growth scanning and estimated fetal weight, it was classified under “growth screening.” Studies focusing on the heart and peripheral vessels were categorized under the “cardiovascular system,” while those addressing intracranial structures and the spinal cord were classified under the “neurological system.” Research on fetal facial structures was labeled as “fetal facial assessment,” and studies on placental evaluation were categorized under “placenta.” Diagnosis and screening of congenital genetic disorders were classified under “genetic disorders,” and studies on the lungs and thoracic structures were categorized under the “respiratory system.” Evaluations of amniotic fluid were classified as “amniotic fluid index,” and research on the fetal urinary system or external genital system was categorized under the “urogenital system.” Any other studies that did not fit into these categories were labeled as “other.”

### Statistical analysis

In the statistical analysis, frequencies (n) along with percentages (%) were used for categorical variables.

## Results

A total of 382 articles on the use of artificial intelligence in perinatology were reviewed. The most cited article in this field, published in 2015 with 251(based on WoS data) citations, is “Standard Plane Localization in Fetal Ultrasound via Domain Transferred Deep Neural Networks” (3). Six articles received over 100 citations each.

**FIGURE 2:**
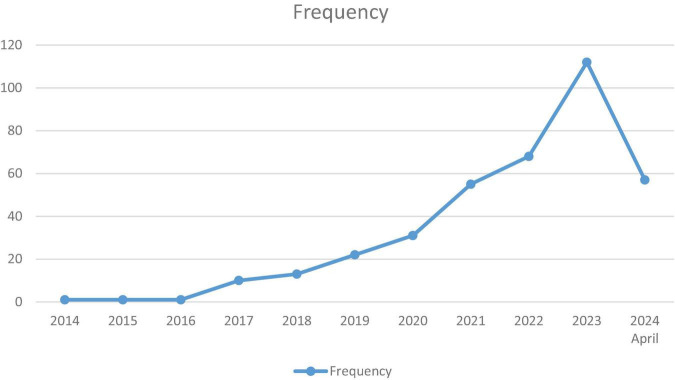
The distribution of the number of published articles on the use of artificial intelligence in perinatology over the years.

**FIGURE 3:**
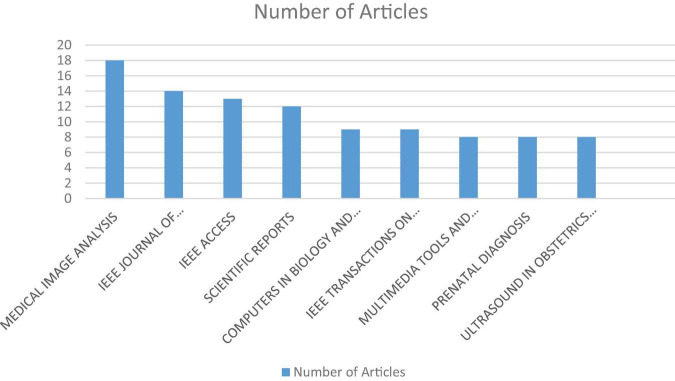
The distribution of articles on the use of artificial intelligence in perinatology by the journals in which they were published. 1-MEDICAL IMAGE ANALYSIS,2-IEEE JOURNAL OF BIOMEDICAL AND HEALTH INFORMATICS,3-IEEE ACCESS,4-SCIENTIFIC REPORTS,5-COMPUTERS IN BIOLOGY AND MEDICINE,6-IEEE TRANSACTIONS ON MEDICAL IMAGING,7-MULTIMEDIA TOOLS AND APPLICATIONS,8-PRENATAL DIAGNOSIS,9-ULTRASOUND IN OBSTETRICS & GYNECOLOGY.

The earliest article encountered in the literature review was from 1995. However, a significant focus on artificial intelligence began to emerge in 2017. In 2023, over 100 articles were published on this topic (Figure 2). Most of the journals where these articles are published are outside the medical field. The journal that shows the most interest in this field is “*Medical Image Analysis*,” followed by the “*IEEE Journal of Biomedical and Health Informatics*” in second place. However, among the top 9 journals that prioritize the use of artificial intelligence in perinatology, there are two medical journals: “*Perinatal Diagnosis*,” which ranks 8th, and “*Ultrasound in Obstetrics and Gynecology*,” which ranks 9th (Figure 3).

Elsevier ranks first among publishing organizations (*n* = 64). This is followed by IEEE-Institute of Electrical and Electronics Engineers Inc (*n* = 47), Wiley (*n* = 47), and Springer (*n* = 44).

### Bibliometric co-occurrence analysis

**FIGURE 4:**
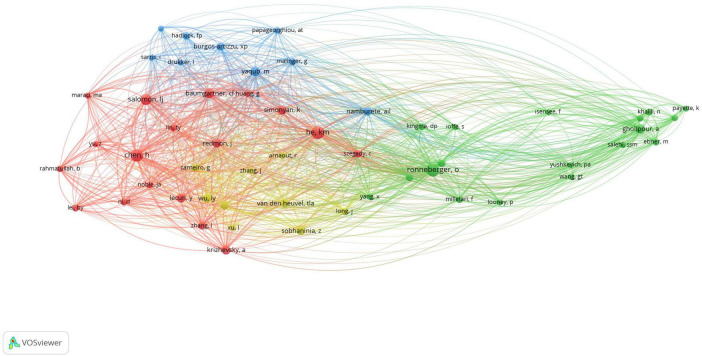
This figure shows the connections between keywords based on how often they appear together in articles. Each point, with different colors and sizes, represents a keyword. A larger point means the keyword is used more frequently. A line between two points shows that both keywords were mentioned in the same article. This map was created using VOSviewer software (version 1.6.19).

A bibliometric co-occurrence analysis was performed on 1,064 keywords extracted from the studies in this list. Keywords with a minimum of five occurrences were included in the analysis, resulting in 46 qualifying keywords. The analysis and visualization were conducted using VOSviewer software, where each keyword is represented by a circle. Keywords that co-occur more frequently are depicted with larger circles. The most frequently used keywords were “deep learning” (*n* = 115), “machine learning” (*n* = 43), and “artificial intelligence” (*n* = 37). The top ten keywords also included convolutional neural network, ultrasound, segmentation, fetal ultrasound, pregnancy, convolutional neural networks, and prenatal diagnosis (Figure 4).

### Bibliometric co-citation analysis

**FIGURE 5:**
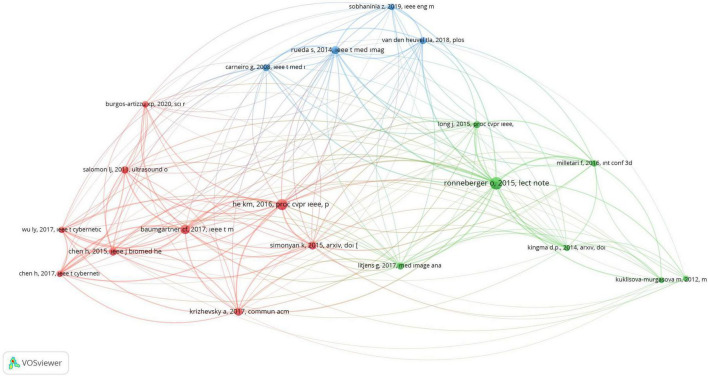
This figure shows the connections between authors based on how often they are cited together. Different colors represent different authors. A larger point indicates that the author has been cited more frequently. A line between two points shows that both authors were cited in the same article. The shorter the line, the closer the relationship between the authors. This map was created using VOSviewer software (version 1.6.19).

A bibliometric co-citation analysis of authors was conducted, and the results are displayed in Figure 5. In the visualization, the proximity of two authors and the thickness of the line connecting them indicate the strength of their co-citation relationship. A line between two authors means they have been cited together in the same article. The analysis included authors with a minimum of three citations, resulting in 54 authors meeting the threshold out of a total of 8,049 authors. Notably, He km. “England” emerged as the most frequently cited author, with a total of 94 citations. Subsequently, Ronneberger O. from “Germany” (*n* = 92), Chen H. from “China” (*n* = 86), Salomon LJ. from “France” (*n* = 68), and Gholipour A. from “Iran” (*n* = 49) were identified, respectively (Figure 5).

**FIGURE 6:**
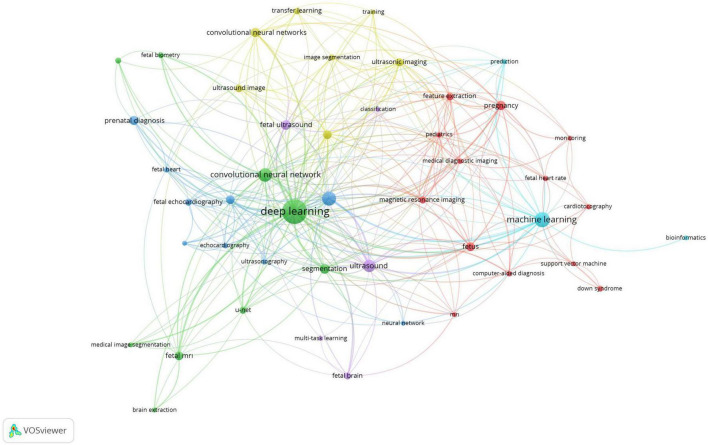
This figure shows the connections between references based on how often they are cited together. Different colors represent different articles, grouping them into clusters. A larger point means the article is cited more frequently. A line between two points shows that both references were cited in the same article. The shorter the line, the closer the relationship between the articles. This map was created using VOSviewer software (version 1.6.19).

Co-citations of references are useful for evaluating the similarity of research by identifying how often these references are cited together across different articles. For this analysis, the minimum citation threshold for a referenced article was set at twenty, resulting in 20 references meeting the criterion out of 10,667 cited references. Notably, the article titled “U-Net: Convolutional Networks for Biomedical Image Segmentation” by Ronneberger O. was the most frequently cited, being referenced in 84 out of the 382 included articles (4) (Figure 6).

### Evaluation of articles with 10 or more citations

The primary application area of artificial intelligence is in screening, accounting for eighty-two articles (67.7%). Thirty-five articles (28.9%) focused on the field of perinatal diagnosis, while only four (3.3%) articles were published in the field of perinatal treatment (Table 1).

**TABLE 1:** The articles with 10 or more citations are categorized and analyzed under the headings purpose of evaluation, medical methods and parameters used, output to be evaluated, and fetal system or region being evaluated with the distribution of these categories examined.

	Number of articles (n)	Percentage of article (%)
**Purpose of evaluation**		
Prenatal screening	82	67.7
Prenatal diagnosis	35	28.9
Prenatal treatment	4	3.3
**Medical methods and parameters used**		
Ultrasound	73	59.8
Magnetic resonance imaging (MRI)	25	20.5
Genetics testing	3	2.5
Electronic fetal monitoring	4	3.3
Physical examination and history	2	1.6
Laboratory blood parameters	5	4.1
Physical examination and laboratory results combination	5	4.1
Fetoscopic surgery	4	3.3
**Output to be evaluated**		
Biometry	34	27.9
Congenital anomaly	17	13.9
Genetic anomaly	8	6.6
Placenta examination	6	4.9
Fetal wellbeing	6	4.9
Organ screening	35	28.7
Fetal prognosis prediction	10	8.2
Teratogenicity	2	1.6
Other	3	2.5
**Fetal system or region being evaluated**		
Growth screening	35	28.7
Cardiovascular system	18	14.8
Neurological system	33	27.0
Fetal facial assessment	2	1.6
Placenta	9	7.4
Genetic disorder	8	6.6
Respiratory system	4	3.3
Other	10	8.2
Amniotic fluid index	1	0.8
Urogential system	1	0.8

Among the materials and parameters where artificial intelligence is used, ultrasound ranks first with 59.8%, followed by MRI at 20.5%. In total, 80.3% of the articles were related to ultrasound or MRI, while the remaining articles were distributed among other categories. Additionally, four articles (3.3%) were in the field of fetoscopic surgery, representing the four articles published in the field of perinatal treatment.

Artificial intelligence is primarily focused on fetal organ screening, accounting for 28.7%. Following this, fetal biometry is the next major focus, with 27.9%. Following that, studies have been conducted using artificial intelligence, the recognition of congenital anomalies (13.9%), and the prediction of fetal prognosis (8.2%).

The systems or region being evaluated are primarily growth screening at 28.7%, followed by the neurological system at 27.0%, and the cardiovascular system at 14.8%.

In studies conducted on ultrasound, the use of artificial intelligence (AI) has primarily focused on fetal biometry recognition, with a particular emphasis on fetal growth screening. In research involving MRI, AI has been applied to the recognition of standard sections used in organ scanning, with an additional focus on the neurological system. Data on other applications of AI in perinatology, including the systems or areas of focus, are presented in Tables 2, 3.

**TABLE 2:** Distribution of the medical methods and parameters used according to the output to be evaluated.

	The output to be evaluated										
		**Biometry**	**Congenital anomaly**	**Genetic anomaly**	**Placenta examination**	**Fetal wellbeing**	**Organ screening**	**Fetal prognosis prediction**	**Teratogenicity**	**Other**	**Total (n)**
The medical methods and parameters used	Ultrasound	33	9	0	2	3	23	2	0	1	73
	Magnetic resonance imaging	1	6	0	1	0	12	3	0	2	25
	Genetics testing	0	0	3	0	0	0	0	0	0	3
	Electronic fetal monitoring	0	0	0	0	3	0	1	0	0	4
	Physical examination and history	0	0	0	0	0	0	1	1	0	2
	Laboratory blood parameters	0	2	2	0	0	0	1	0	0	5
	Physical examination and laboratory results combination	0	0	3	0	0	0	1	1	0	5
	Fetoscopic surgery	0	0	0	3	0	0	1	0	0	4
	Total (n)	34	17	8	6	6	35	10	2	3	121

**TABLE 3:** Distribution of the medical methods and parameters used according to the fetal system or region being evaluated.

	The fetal system or region being evaluated											
		**Growth screening**	**Cardiovascular system**	**Neurological system**	**Fetal facial assessment**	**Placenta**	**Genetic disorder**	**Respiratory system**	**Other**	**Amniotic fluid index**	**Urogential system**	**Total (n)**
The Medical methods and parameters used	Ultrasound	33	13	12	2	2	0	2	7	1	1	73
	Magnetic resonance imaging	1	0	20	0	3	0	1	0	0	0	25
	Genetics testing	0	0	0	0	0	3	0	0	0	0	3
	Electronic fetal monitoring	0	4	0	0	0	0	0	0	0	0	4
	Physical examination and history	1	0	0	0	0	0	0	0	0	0	2
	Laboratory blood parameters	0	1	1	0	0	2	1	1	0	0	5
	Physical examination and laboratory results combination	0	0	0	0	0	3	0	2	0	0	5
	Fetoscopic surgery	0	0	0	0	4	0	0	0	0	0	4
	Total (n)	35	18	33	2	9	8	4	10	1	1	121

## Discussion

Through bibliometric analysis, I witness the rising influence of artificial intelligence (AI) in the field of perinatology. The exponential increase in publications related to AI in perinatology since 2017 is evidence of this. I am witnessing a historical transformation in this field. The fact that I has recorded 382 articles in this area to date is an indicator of a nascent trend. The rate of increase in articles published between 2022 and 2023 suggests that AI applications may become central to perinatology in the coming years (Figure 2).

Among the top-ranking journals where these articles were published, two medical journals have emerged as significant contributors. These journals, known for their high impact factors, hold substantial influence in the field of perinatology. The top seven journals, as expected, primarily consist of engineering journals. *Medical Image Analysis* has been the journal with the highest number of publications in the field of artificial intelligence in perinatology (*n* = 18). The main reason for this could be that, as noted in the study data, 80.3% of the publications focus on medical imaging systems. *The IEEE Journal of Biomedical and Health Informatics* (*n* = 14) and *IEEE Access* (*n* = 13) have emerged as two other prominent journals publishing in the field of perinatology. *Scientific Reports* ranks fourth with 12 articles. This journal not only covers topics related to artificial intelligence in perinatology but also addresses AI in other medical fields, and it has high citation rates (Figure 3) (5).

It has been revealed that non-medical journals have shown greater interest in artificial intelligence applications in the field of perinatology. In particular, journals in the engineering field have demonstrated more interest in this area and have allocated space for such studies. The primary reason for this is attributed to the integration of artificial intelligence applications into fetal imaging techniques, such as ultrasonography and MRI, which require engineering expertise. This study found that over 80% of the articles focused on fetal imaging techniques. While this is an expected outcome, it can be observed from the data that such studies are relatively underrepresented in perinatology journals. As one of the conclusions of this paper, it can be suggested that medical journals should allocate more space to articles designed around artificial intelligence applications in the field of perinatology.

There are two medical journals in the field of perinatology that feature a similar number of articles.

The journal *Prenatal Diagnosis*, which ranks 8th, has an Impact Factor (IF) of 3.24 (2021). Notably, this journal has observed the rising influence of AI in perinatology and allocated space for related studies. Its most cited publication, with 20 citations, is “Noninvasive screening for congenital heart defects using a serum metabolomics approach” (6).

Ranked 9th, *Ultrasound in Obstetrics and Gynecology* is a journal with significant influence in the field of obstetrics and gynecology. The journal’s IF has been recorded as 8.6 (2021). The most cited paper from this journal, published in 2020 with 61 citations, is “Using deep-learning algorithms to classify fetal brain ultrasound images as normal or abnormal” (7).

Research that began in the field of perinatal screening has continued to expand, evolving into studies concerning perinatal diagnosis and even perinatal treatment. Among the evaluated articles with 10 or more citations, 67.7% were focused on fetal screening. The primary goal in screenings is to recognize standard measurements and facilitate the acquisition of automatic measurements. The first priority in this regard is the acquisition of standard plans for organ screening, followed by the automation of fetal biometry. A 2022 article titled “No sonographer, no radiologist: New system for automatic prenatal detection of fetal biometry, fetal presentation, and placental location” supports this claim (8). It asserts that accurate screenings can be performed independently of sonographers and radiologists.

Only four articles have been found to focus on the application of perinatal treatments. All the articles published in this field were about the application of fetoscopic surgery in twin pregnancies. The most cited article in this area, with 18 citations, was published in 2021 and titled “A shape-constraint adversarial framework with instance-normalized spatio-temporal features for inter-fetal membrane segmentation” (9). This publication appeared in the journal “Medical Image Analysis,” which is also one of the top three journals making significant contributions to this field. In our literature review included in the study, three additional articles related to prenatal treatment were identified (10–12).

In the field of perinatology, artificial intelligence applications have been studied extensively, especially to develop imaging systems. As expected, ultrasound predominates, focusing primarily on automating standard measurements. The goal is to enhance patient evaluation and ensure adherence to specific standards independent of the expertise of ultrasound or radiology specialists. Among the top five most cited articles in this area, three focus on creating standard plans. Topping the list with 259 citations is the 2012 article titled “Standard Plane Localization in Fetal Ultrasound via Domain Transferred Deep Neural Networks” (3). Following closely with 209 citations is the 2017 paper titled “SonoNet: Real-Time Detection and Localization of Fetal Standard Scan Planes in Freehand Ultrasound” (13). Rounding out the list is the 2021 article with 120 citations titled “Automatic Fetal Ultrasound Standard Plane Recognition Based on Deep Learning and IIoT” (14). The primary objective of ultrasound examinations has been the assessment of biometry and fetal growth. The secondary focus has been on the fetal cardiovascular system, followed by the neurological system. It is known that congenital anomalies of the neurological system are the most common congenital anomalies (15). Additionally, cardiovascular system anomalies are also quite frequent, and prenatal diagnosis has a positive impact on postnatal prognosis (16). Therefore, prenatal diagnoses of these two systems are important in perinatology. The focus of artificial intelligence on these systems can be interpreted as a response to this need. From another perspective, it is evident that 13 out of 18 publications focused on the cardiovascular system have applied artificial intelligence to ultrasound. Therefore, it can be concluded that artificial intelligence applications in the evaluation of the fetal cardiovascular system are predominantly conducted through ultrasound.

The next most common application of artificial intelligence after ultrasound has been in MRI imaging. Among the 121 most frequently cited publications, 25 focused on MRI. Interestingly, 20 of these publications were related to the fetal neurological system. The frequent use of fetal MRI in assessing the fetal neurological system may have established a specific domain for artificial intelligence applications in this area.

According to the bibliometric co-citation analysis, the most frequently cited authors have been identified. When examining the institutions where these authors work, it is evident that research is not confined to a single region but spans across different areas. The fact that studies on the effects of artificial intelligence in perinatology come from such diverse regions can be seen as an indicator of the field’s growing popularity. Moreover, this trend can be interpreted as a sign that artificial intelligence will become even more popular in perinatology in the coming years. The increasing number of publications over time clearly supports this. In the future, I can expect AI to have an even greater impact on perinatology.

The most frequently used keyword was “deep learning,” and it was significantly more prominent than other commonly used keywords. This finding may help our colleagues who are researching this topic to keep “deep learning” in mind as a key term. In literature searches and article writing, using the term “deep learning” could be a useful strategy for accessing more publications.

There are two main potential sources of bias in the study.

The first is the lack of controlling for authors in connectivity analyses, which is a significant source of bias. Certain research groups tend to use specific keywords, and many authors or research groups frequently cite within their own work, further amplifying this issue. However, these points may not be fully clarified through analyses conducted via the WoS system.

The second is the study’s reliance solely on articles indexed in the WoS system. Full texts of all articles obtained through this system were accessed. However, it is true that searches conducted using specific keywords may have excluded some articles outside the system. Nevertheless, given the large sample size, it is believed that the findings derived from this study accurately reflect general trends.

In conclusion, I has witnessed the increasing influence of artificial intelligence in the field of perinatology in recent years. This impact may mark the historic beginning of the transition to the AI era in perinatology. Milestones are being laid on the path from prenatal screening to prenatal treatment.

## Data Availability

The raw data supporting the conclusions of this article will be made available by the authors, without undue reservation.
